# Supplementary material for: Are carbon emissions trading and green financial instruments synergistic? -Comprehensive quantitative research based on content analysis
Source: PLoS One. 2024 Mar 7;19(3):e0298601. doi: 10.1371/journal.pone.0298601 (PMC10919601; doi:10.1371/journal.pone.0298601)
Supplement: S1 Data — (ZIP) [file pone.0298601.s001.zip › Data/地级市Z_F工作报告.zip!/╝¬┴╓/╦╔╘¡╩╨/└┤╫╘╣1⁄2╓┌║┼í╛┬φ┐╦╩2╛▌═°í┐_2018.docx]

各位代表：

　　现在，我代表市人民政府向大会报告工作，请予审议，并请市政协各位委员提出意见。

　　一、2017年工作回顾

　　过去一年，是本届政府开局之年，也是松原发展史上很不平凡的一年。面对严峻复杂的经济形势、前所未有的困难挑战、艰巨繁重的工作任务，我们在省委省政府和市委的坚强领导下，团结带领全市各族人民，紧紧围绕生态振兴、转型崛起，坚定不移抓住“一个统领”，聚精会神建设“两个城市”，全力以赴打好“三大战役”，沉着应对困难和挑战，妥善处理矛盾和问题，砥砺奋进、合力攻坚，取得了来之不易的发展成果。预计（下同）全市地区生产总值实现1706亿元，同比（下同）增长1%左右。地方级财政收入完成47.9亿元，下降5.6%。全社会固定资产投资完成1437亿元，增长1.5%。社会消费品零售总额实现715亿元，增长7%。城乡常住居民人均可支配收入分别达到32688元和10870元，均增长6%。主要做了以下工作：

　　（一）紧扣提质、逆势前行，工业经济稳中向好。新增规模以上工业企业20户；工业增加值实现640亿元，增长2%左右。我市成功入选全国首批产业转型升级示范区。扶余工业集中区被纳入全省开发区体制机制创新试点。主导产业持续壮大。全力支持吉林油田公司稳油增气，油气产量当量达到470万吨。松原石化10万吨丙烯一期、吉星日产50万立方米LNG项目建成投产。金海石化5500吨苯乙烯阻聚剂项目建设完工，填补全省该类产品生产空白。天船马铃薯综合开发利用项目投入生产。鲁花10万吨花生油项目主体完工。嘉吉生化200万吨玉米深加工扩能、晰晰农业马铃薯种植及深加工等项目开工建设。优势产业态势强劲。信诚爱谷50万吨绿色蔬菜产业园及有机肥、中盐红四方肥业二期、云天化20万吨钾肥项目建成投产。新研牧神农机扩能、殴彼龙汽车轮胎及防冻液项目投产达效。新兴产业培育加快。黄栀花药业口服液、吉隆生物药业变性淀粉项目建成投产。新创生化总溶剂项目重新启动。阳光凯迪年产20万吨生物质合成油项目完成部分桩基础工程。顺泰生物质热电联产项目主体完工。长岭500千伏超高压输变电站进入设备调试阶段，为输出我省西部地区过剩电能奠定了良好基础。

　　（二）紧扣绿色、集约增效，现代农业发展提速。一产增加值实现277亿元，增长3.5%。战胜低温、干旱、内涝等灾害，粮食总产量达到744.5万吨。围绕基础设施强农业。哈达山水利枢纽工程收尾、松原灌区工程顺利推进，华侨农场灌区项目完成建议书编制，引松入扶二期工程开工建设，河湖连通项目完成前期工作，西部土地开发整理圆满收官，新增高标准农田22万亩。围绕绿色有机强农业。以农业供给侧结构性改革为突破口，致力于打造绿色农业城，新建绿色有机农业示范基地41个，“三品”认证面积突破300万亩，“善德良米”被确定为厦门金砖国家峰会指定用米，扶余市获批国家级出口食品农产品质量安全示范区。“我在松原有一亩田”项目顺利推进，22个省份消费者认领土地6000亩，松原农产品加速走向全国人民的餐桌。围绕集约高效强农业。调减籽粒玉米种植面积78万亩，新增瓜果、棚菜、食用菌、黑玉米、中药材等特色作物种植和稻田养蟹面积96万亩，乾寿现代果蔬产业园建成温室大棚240栋，乾安县获批国家级农村产业融合示范区。着力推进规模化、集约化、标准化经营，天丰谷物黄小米等3个种植区获批省级农业标准化示范区，扶余绿兴等3个农民合作社被评为国家级示范社，新增新型农业经营主体1400个，流转土地353万亩。围绕精品养殖强农业。正邦200万头生猪养殖加工一体化三期、大北农80万头生猪养殖项目建成投产，华西希望200万头生猪养殖项目扎实推进，齐全100万头生猪繁育及有机肥料加工三期、牧原100万头生猪养殖项目开工建设，科尔沁万头肉牛养殖扩建项目基本建成。新建标准化规模养殖场（小区）170个。

　　（三）紧扣升级、多点发力，服务业活力持续释放。服务业增加值实现746亿元，增长5.1%。旅游业发展势头强劲。查干湖冰雪渔猎文化旅游节获评“国家级示范性渔业文化节庆”。查干湖屯被农业部等部委评为“中国十大最美乡村”之“最美渔村”。查干南湖水产品展销中心主体完工，大汗蒙古风情园改造基本完成。查干湖生态文化旅游特色小镇项目完成前期工作。威尼斯水上乐园一期完成主体封闭。绿动松原项目全面启动。商贸物流业发展向好。东镇商业广场投入运营，大润发超市江北店完成主体工程。星火医药物流园跨入省级现代服务业集聚区行列，敬亿国际商贸物流城一期、尚谷粮食现代物流园投入运营，史丹利30万吨粮食仓储物流项目完成升级改造，瑞禾仓储物流园三期冷库项目全面建成，郭尔罗斯农副产品物流产业园开工建设。房地产业稳中有进。完成投资25亿元，开发面积110万平方米，销售商品房61万平方米。金融业发展步伐加快。吉林股权交易所松原运营中心投入运行，新力源化工等71户企业在吉林股权交易所展示板和精选板挂牌。吉林省物权融资农业发展公司松原分公司挂牌成立。光大银行松原分行开业运营。人保健康寿险公司落户松原。农村金融综合改革试点深入开展，农牧贷、农机贷、大棚贷等系列金融产品创新成果显著。信息服务等产业发展加速。“数字松原”软硬件建设基本完成，云计算大数据中心建设全面启动，守望乡村东北电商园蓬勃发展，“扶余四粒红花生”大数据信息应用平台建成运营，全省首个农村淘宝3.0模式试点效果明显，松云电子科技公司组建成立。连锁经营、文化创意、健康养生等服务业态发展加快。

　　（四）紧扣实效、狠做实功，招商引资和项目建设成效显著。招商引资成果喜人。北京松原商会和广东松原商会完成组建，成功举办松原—北京投资说明会暨项目签约仪式等系列招商活动，襄大百万头生猪全产业链、兴能镍氢动力电池等一批“四高”项目相继签约。积极参加东博会、吉商大会等各类招商活动，签约德义半导体产业园、上海超导第二代高温超导带材、科莱达3D制板绿色建筑等重大项目108个。广泛与舟山等地开展战略合作，成功举办松原—舟山城市旅游推介会等活动，分别与舟山市政府和舟山商贸集团、杭萧钢构公司等签署战略合作协议。全年招商引资到位资金1034亿元，增长10%。项目建设高效推进。深入落实全省“三抓”“三早”行动部署，有效实施“四抢”行动计划，开复工5000万元以上项目151个，完成固定资产投资227亿元。64个项目纳入省PPP项目库，数量居全省首位。

　　（五）紧扣宜居、做优生态，城乡建设统筹推进。中心城市建设扎实推进。我市成功列为国家重大市政工程领域PPP创新工作重点城市和全省城乡垃圾收转运体系试点城市。城市供排水、停车场等专项规划完成编制。源江路、嘉禾街、建设街等6条街路新建续建项目全部竣工。松原大路、文化路等路段地下综合管廊工程有序推进。天河大桥南北岸互通项目进入设计阶段。海绵城市一期工程进入社会资本采购阶段。无名泄支黑臭水体整治和圣湖补水工程开工建设。江南、江北城区污水深度处理项目投入运行。新增市城区集中供热联网面积25.5万平方米。新建市城区燃气管网10公里。中山大街、沿江路等13处市政工程和公园、广场完成绿化彩化，新增绿地面积20万平方米。东镇大路、滨江大道等街路及周边楼体亮化全面升级。扎实开展市容环境综合整治“十大战役”和综合整治月活动，城市管理和环境卫生明显改善。立体化交通格局基本形成。松原查干湖机场正式通航，通达8个大中城市，架起连接域外的“空中走廊”。长白乌城际快铁投入运行。长西铁路续建项目顺利推进。松乾高速项目征地拆迁全面启动，京哈高速长春至拉林河段、铁科高速榆树至松原段完成征地拆迁，长太高速项目进入招标阶段。松原火车站站前综合改造和宁江客运站完成主体工程。新农村建设取得积极进展。建成美丽乡村7个。绿化美化省县两级示范村屯207个。新建农村厕所15428个。建设农村公路330公里。植树造林17.2万亩，义务植树263万株。地震灾区建成崭新家园。投入救灾资金6700万元，疏散并妥善安置群众8000余人，新建、修缮和加固房屋3632户，地震灾区所有房屋受损户入冬前全部住进了安心、暖心的房子。生态环境更加绿色宜居。获批国家电能替代试点城市。购置运营新能源公交车146辆。全面贯彻落实河长制，松花江干流治理工程全面完成，饮用水水源地规范化建设全面开展，实施排污许可证制度并走在全省前列。规范治理加油加气站306座，淘汰市建成区10蒸吨以下燃煤小锅炉92台，强力推进秸秆禁烧，城市空气二级以上优良天数达到289天。治理规模化养殖场（小区）788个。

　　（六）紧扣改革、合力攻坚，内生动力不断增强。供给侧结构性改革有效推进。全面落实“三去一降一补”任务，去除玉米库存314万吨，整治和拆除“地条钢”企业2户，清退僵尸企业635户，房地产企业、中小企业去杠杆有效推进。“放管服”改革扎实推进。削减行政审批事项19项，清理规范行政审批中介服务事项26项。动态管理权力清单、责任清单和财政专项资金清单，企业投资项目管理负面清单全面建立。深入推进职业资格改革，扎实开展违规职业资格清理专项行动。农业农村改革全面推进。农村集体产权制度改革试点、农村棚膜设施确权颁证试点深入推进。农村土地和查干湖水面经营权确权登记全面完成。农村集体建设用地和房屋调查确权登记外业调查基本结束。供销合作社综合改革、国有林场改革和农垦改革取得积极进展。农村就业制度综合改革试点启动实施。行政事业单位改革有序推进。公务用车制度改革、机关事业单位养老保险并轨认定圆满完成。行政职能事业单位改革试点顺利实施，生产经营类事业单位改革在全省率先完成。城市管理执法体制改革试点稳步推进。市机关事务管理局、市公共资源交易中心、市重大项目服务中心组建成立。此外，文化市场综合执法、公交车公有公营、盐业监管等领域改革顺利推进，城市公立医院改革全面铺开，财政国库集中支付实现电子化，吉林油田公司供水、供电业务完成移交。

　　（七）紧扣精准、求真务实，脱贫攻坚取得阶段性成果。坚持精准聚焦、精准发力，104个贫困村实现脱贫摘帽，21294名贫困群众告别贫困。基础短板不断补齐。完成建档立卡贫困户危房改造8493户，改造低压电网线路355公里，完成190个村屯饮水安全巩固提升工程。产业脱贫持续发力。投资2.8亿元，实施棚膜建设、特色种植养殖等富民产业项目234个，齐全“四六开”托养、屋顶分布式光伏发电等项目有效实施。行业扶贫成效显著。向家庭经济困难学生发放助学资金1150万元。给予13230名贫困群众新农合保险金减免待遇。免费为300名贫困残疾群众实施白内障复明手术。组织20户民营企业与贫困村开展结对帮扶。发放扶贫小额贷款2000万元。开展各类技能培训，实施促就业“春风行动”，帮助7200名困难群众实现创业就业。

　　（八）紧扣共享、增进福祉，社会建设全面加强。保障和改善民生深入推进。大力实施民生行动计划，全力办好各类民生实事，人民群众获得感不断增强。新增城镇就业4.6万人，完成农村劳动力职业技能培训1.49万人，转移农村剩余劳动力67万人次。医疗保险实现跨省异地就医直接结算。长期护理保险制度建立实施。养老、失业等社会保险进一步扩面。退休人员养老金、失业保险金、城乡低保金全面提标。“居家+社区+机构”的养老服务体系初步形成。“农村养老大院+”模式有效推广。“情暖松原”系列救助活动覆盖全市。发放住房公积金贷款5亿元。改造棚户区20.8万平方米。油田矿区327万平方米“暖房子”工程全面竣工。土地和房屋不动产产权登记完成52万平方米。市规划区震害预测和市防震减灾科普基地项目全面完工。市救灾指挥中心完成主体工程。市食品药品检验中心主体竣工。社会事业蓬勃发展。教育“九大工程”深入实施。县级义务教育均衡发展顺利通过国家验收。新建重建的5所中小学校舍全部竣工。儿童之家项目进入内装阶段。前进石油机械公司双游动卡瓦装置等获国际发明专利。百年汉克制药公司被认定为国家级高新技术企业。鸿翔种业等4户企业被认定为省级小巨人企业。牧神玉米收获机等10个产品被认定为吉林省名牌产品。新增有效注册商标1567件。市知识产权运营服务体系建成。市中心医院异地新建项目内装基本完工。圆满承办“航空飞镖—2017”国际军事比赛和全省第七届少数民族传统体育运动会。市体育馆投入使用。市冰上运动中心项目完成主体建设。“市标、市树、市花”评选落幕。建立“新时代传习所”2000个。满族新城戏《神鼓传奇》获国家艺术基金扶持。春捺钵遗址群安防工程全面竣工。繁荣等4个村获评全国文明村。郭尔罗斯社区获评全国社区侨务工作示范单位。统计、宗教、外事、气象、水文、人防、档案、红十字、地方志等工作都取得了新成绩。社会治理不断加强。“七五”普法全面启动。人民调解组织实现城乡和行业全覆盖。扎实开展信访“百日攻坚”和市领导接访下访等活动，化解各类信访案件210件，党的十九大召开期间实现进京非访“零登记”、极端事件“零发生”。强力开展安全生产“落责任、守规章、减事故、保平安”等活动，中央和省督察组反馈的安全隐患得到全面有效整改。立体化治安防控体系建设不断加强，“三打击一整治”专项行动和“五个攻坚战”有力开展，成功破获非法种植加工贩卖大麻等大案要案。全面提高应急处突能力，妥善处置“7·04”燃气爆燃等各类突发事件，社会大局保持和谐稳定。

　　各位代表！过去一年，我们着力加强政府自身建设。扎实推进“两学一做”学习教育常态化制度化。自觉接受市人大及其常委会法律监督、工作监督和市政协民主监督，认真办理市人大代表建议、政协提案231件。全面加强政务公开和政务服务“一张网”建设，不断强化行政监察和审计监督，公职人员服务意识和机关行政效能进一步增强。

　　各位代表！过去一年，在困难集中显现、风险相互交织的形势下，我们顶住了经济下行的压力，排除了前进道路上的障碍，开创了转型发展的新局面，“稳”的基础更加牢固，“进”的态势正在向好，各项成绩来之不易！这些成绩的取得，是省委省政府和市委统揽全局、坚强领导的结果；是市人大、市政协有效监督、鼎力支持的结果；是在历届政府和老领导、老同志打下的坚实基础上，全市广大干部群众团结拼搏、奋发进取的结果；是中省直单位、驻松部队、武警官兵、消防战士和社会各界群策群力、携手共建的结果。在此，我代表市人民政府，向所有为松原发展建设作出贡献的同志们、朋友们，致以崇高的敬意和衷心的感谢！

　　各位代表！在看到成绩的同时，我们也清醒地认识到，工作中还存在不少困难和问题，主要表现为：经济下行态势比预期更加严峻，原油量价低位徘徊对经济发展的影响持续加大；转型发展面临严重制约，产业升级依然任重道远；高质量发展意识不强，抓投资、上项目、优先发展产业项目的思想树得不牢；财政收支矛盾加剧，城乡居民收入增长不快；保障民生仍需加强，脱贫攻坚任务繁重；资源环境约束加大，生态改善任务艰巨；安全生产形势严峻，责任落实有待加强；营商环境不尽人意，行政效能低、企业办事难等问题尚未得到根本解决；部分干部思想不够解放，一些部门执行力不强，个别公职人员不作为、乱作为和不敢担当、不愿担当等问题依然比较突出。对此，我们高度重视，将在今后工作中认真加以解决。

　　二、2018年主要工作

　　2018年是贯彻党的十九大精神的开局之年，是决胜全面建成小康社会、实施“十三五”规划承上启下的关键一年。当前，松原正处于转型发展最艰难、最要劲的关键时期，正处于负重进阶的阵痛期和不进则退的临界点上。新的一年，我们必须进一步增强机遇意识、忧患意识、担当意识，保持定力、坚定信心，以更大的勇气和智慧开拓前行，以更实的作风和举措拼搏进取，努力在新的起点上谋求新发展、实现新突破、开创新局面。

　　做好今年政府工作的总体要求是：深入贯彻落实党的十九大和省“两会”、市委六届四次全会精神，高举习近平新时代中国特色社会主义思想伟大旗帜，按照统筹推进“五位一体”总体布局、协调推进“四个全面”战略布局和深入实施“三个五”战略要求，牢固树立和贯彻新发展理念，以继续深化供给侧结构性改革为主线，紧扣生态振兴、转型崛起，抓住“一个统领”不动摇，建设“两个城市”不放松，打好“三大战役”不懈怠，聚焦提升发展质量、深度开发资源、放大比较优势、抓实产业项目、培育壮大产业，全面做好稳增长、调结构、促改革、惠民生、防风险各项工作，高水平谱写全面建成小康社会的松原篇章。

　　按照这个总体要求，今年全市经济社会发展的主要预期目标是：地区生产总值增长5.5%，地方级财政收入实现正增长，全社会固定资产投资增长5%左右，社会消费品零售总额增长7%，城乡常住居民人均可支配收入分别增长6%和8%，城镇登记失业率控制在4.5%以内。重点做好以下工作：

　　（一）转型升级稳工业。坚持转变方式与优化结构并举，扩大总量与提升质量并重，着力推进工业经济坚实增长。深入挖掘油气经济潜力。全力支持吉林油田公司、东北油气公司遏制原油减产态势，力争油气产量当量达到462万吨。全面完成“四供一业”资产分离移交，助推吉林油田公司减负发展。推动瑞兴化工二硫化碳项目建成投产，加快推进众诚集团10万吨油页岩原位裂解中试项目，开工建设中科聚合500吨聚芳醚酮项目。加快发展壮大非油工业。着力发展农产品精深加工业，积极推进玉米全产业链开发和杂粮杂豆等作物加工转化，规划建设嘉吉综合产业园，加快推进盼盼食品产业园建设，完成吉安生化、天源生化重整并实现投产，全力推动鲁花10万吨花生油、晰晰农业马铃薯种植及深加工、汇海酒业5万吨酒精等项目建成投产，加快广骥玉米休闲食品等项目建设进度，积极推进正轩水果玉米饮料及罐头等项目。着力发展生物医药和生物化工产业，支持百年汉克、和元生物制药等企业做大做强，开工建设黄栀花药业中药材提取项目；推动新创生化公司全面复产，加快推进阳光凯迪年产20万吨生物质合成油项目。着力发展新材料和化肥产业，开工建设禾木之家200万立方米生物质建筑材料项目；推动德润20万吨掺混肥、佳禾30万吨有机肥、澳佳50万吨腐殖酸功能肥等项目建成投产，促进心连心60万吨复合肥、云图控股复合肥项目签约落地。着力发展装备制造和加工业，加快推进两江高科技产业孵化园建设，开工建设中科惠锋70万片晶片光电材料制造基地项目；完成海川纸业扩能改造，推动山鹰纸业百万吨工业用纸项目落地建设。

　　（二）集约增效强农业。按照产业兴旺、生态宜居、乡风文明、治理有效、生活富裕的总要求，启动实施乡村振兴三年攻坚行动，深入推进农业供给侧结构性改革，加速打造全国知名的绿色农业城。着力提升农业综合生产能力。加快推进松原灌区、河湖连通、引松入扶二期、花敖泡蓄水调蓄等重点工程建设。新增高标准农田24万亩、高效节水灌溉24.8万亩。正常年景粮食总产量达到750万吨。着力发展现代农业。大力推进“互联网+农业”和智慧农业发展。积极推进海峡两岸（吉林）生态农业合作先行实验区、农业循环经济和玉米膜下滴灌农业标准化示范区建设。开工建设全省首个马铃薯种薯组培实验室。土地规模化经营面积达到450万亩。着力打响农产品品牌。依托“我在松原有一亩田”项目，大力提高“查干湖大米”“善德良米”“乾安黄金小米”等品牌的知名度和美誉度，以品牌抢占中高端市场。着力调整优化种植结构。持续建好绿色农业示范基地。新增“三品”作物种植面积20万亩。大力发展瓜果、棚菜、食用菌、黑玉米、中药材等特色作物种植，酿酒红高粱种植面积达到3万亩，油用牡丹种植面积达到4000亩。积极推广稻鱼、稻蟹、稻鳅等种养模式，新增种养面积1.5万亩。着力做强优质畜牧业。推动华西希望200万头生猪养殖、正大300万只蛋鸡全产业链等项目建成投产，加快推进特沃斯牧业万头精品肉牛等重大项目，新建标准化规模养殖场（小区）80个。着力加强新农村建设。全面推进“美丽乡村”建设。新建美丽庭院2000个、干净人家5000户。绿化美化省县两级示范村屯208个。大力推进“厕所革命”，改造农村厕所1万个。新建农村公路262公里。完成农村广电网络升级改造，实现广电网络光纤全覆盖。

　　（三）提档升级促三产。全力打好服务业发展攻坚战，推动生产性服务业向专业化和价值链高端延伸、生活性服务业向精细化和品质化转变。大力发展特色旅游业。办好查干湖冰雪渔猎文化旅游节、莲花文化旅游节和全国半程马拉松赛等系列活动。深化与海航集团等大企业的战略合作，推动查干湖游客服务中心投入运营，加快推进莲花产业园项目。启动建设查干湖生态文化旅游特色小镇。推动威尼斯水上乐园一期项目投入运营，扎实推进绿动松原等项目建设。大力发展航空服务业。扎实推进查干湖空港经济园项目，规划建设长岭通用航空机场，积极培育航空冷链物流、快递物流、电子商务物流及配送体系。大力发展商贸物流业。实现大润发超市江北店开业运营，开工建设欧丽洛雅商贸综合体。推动沈铁物流园和郭尔罗斯农副产品物流产业园投入运营，改造升级三井子杂粮交易市场，开工建设豪森冷链仓储物流配送二期项目。大力发展房地产业。完善科学供地机制和住房消费鼓励政策，逐步推广装配式建筑，促进房地产业稳健发展。完成投资16.5亿元，开发商品房80万平方米。大力发展金融业。争取更多优质企业在吉林股权交易所挂牌。推动宁江、长岭农信社改制为农商行。扎实开展农村金融综合改革试验，有效推进农村物权融资体系建设，创新林地、草场、水面等资源性物权融资产品，扩大农村土地经营权抵押贷款、农村棚膜设施抵押贷款和土地收益保证贷款范围。创新政府资源配置方式，加大投融资力度。有效管控松发、松农、松旅集团存量债务，加强银行、小贷公司等各类金融主体监管，坚决防范财政和金融风险。大力发展信息和文化创意等服务业。着力推进智慧城市建设，扶持松云电子科技公司做大做强，加快建设县域电子商务产业园及运营中心，支持夫余国传媒公司开展智慧社区服务。加快推进春捺钵遗址保护工程，推动前郭鼎润非遗创意产业园投入使用。积极发展其他新型服务业态。

　　（四）统筹城乡建家园。牢固树立“经营城市”理念，扎实做好征地、拆迁、土地收储和融资等工作，突出生态、彰显宜居，狠抓建设、强化管理，全力打造天蓝、地绿、水清的“高颜值”、高品质花园之城，加快提高城镇化水平。着力增强城市综合承载能力。完成铁南片区、城区农贸市场等专项规划编制。启动建华东路、滨江大道等道路续建、延伸工程。实施文化路、松原大路等14条街路地下综合管廊新建续建工程。推动松原火车站站前综合改造工程全面竣工。争取宁江客运站投入使用。开工建设松湖快速路。积极推进长白乌快铁二期和长西铁路项目。加快长太高速、松乾高速、京哈高速长春至拉林河段、铁科高速榆树至松原段、国道203线肇源至善友段项目建设。扎实推进一江两岸综合开发。深入实施沿江开发战略，启动海绵城市一期建设工程，加快建设百里绿廊，完成天河大桥南北岸互通项目年度建设任务和纳仁汗公园二期改造项目。深度打造一江两岸环境，将其变成一道靓丽的风景线。大力推进城市绿化美化亮化升级，让城市更加美丽、更有品位。有效提升城市管理水平。继续打好市容环境综合整治“十大战役”，保持高压严管态势，深入开展城市出入口等重点区域专项整治。加强道路交通治理，严禁车辆随意停放、马路摆摊、路边烧烤等违规行为。完成智能化城市管理信息指挥中心项目前期工作。持续加强生态文明建设。全力整改中央环保督察反馈问题。有序推进第二次污染源普查。打好污染防治攻坚战，深入实施清洁空气、清洁水体、清洁土壤和国土绿化行动计划。推动城乡垃圾收转运一体化、餐厨垃圾及污泥资源化综合利用项目投入使用。积极推进供热站脱硫脱硝改造，实现市建成区10蒸吨以下燃煤小锅炉全部“清零”，规划建设热电联产项目。扎实做好秸秆禁烧工作。深入推行河长制，实施春季河道清洁整治专项行动。扎实推进圣湖补水工程，加快城市黑臭水体治理，开工建设江南污水处理厂10万吨扩能工程，完成查干湖污水处理厂管网建设并投入使用，推动松林断面水质达到Ⅲ类标准。开展土壤污染状况详查和评估，强化农业面源污染整治，切实抓好畜禽养殖污染防治。加强土地沙化碱化及草原退化等综合治理。

　　（五）内引外联激活力。加快推进基础性、关键性改革，着力构建宽领域、深层次开放格局，有效激活发展动能。全面推进深化改革。理顺市本级教育管理体制。启动环保机构监测监察执法垂直管理体制改革。组建市城市管理行政执法机构。积极推进供水、供热、排污等公用事业实现市场化投资、运营和管理。深入推进国有林场改革和农垦改革。扩大农村集体产权制度改革试点和“粮食银行”改革试点范围。推动全市供销系统综合改革向纵深拓展。高效精准招商引资。牢固树立以项目论英雄、论政绩导向，着力掀起全民招商热潮。锁定京津冀、长三角、珠三角等重点地区，深化与舟山等城市和国内知名科研院所的战略合作，筹划举办优质农产品及精品杂粮展洽会等系列经贸活动，着力引进一批大型领军企业和重大优质税源型项目。推动各县（市区）、开发区着力保障征拆、融资等项目落地要素支撑，全力以赴引项目、落项目、建项目，主要领导必保完成“新签约、新开工、新投产”投资3亿元以上项目各1个目标任务，健全招商激励政策，定期开展项目建设巡检考评，倒逼招商引资取得实效。扶持壮大县域经济。加大对县域发展的支持力度，启动实施县域经济转型升级示范工程，有效落实扩权强县改革要求。推动扶余市纳入全省三次产业融合发展扩权强县试点，着力建成与哈长两市联动发展的产业隆起带；前郭县依托长春、松原发展节点，突出打造吉林西部特色生态旅游、农产品加工转化产业集聚区；长岭县融入长春经济圈，加快建设长春产业转移配套基地；乾安县着力建设绿色弱碱农产品和精品畜牧业生产加工基地，打造“中国杂粮之乡”。统筹推进长山、太平川等特色小城镇建设，大力发展特色经济，增强对产业、人口等吸纳和综合承载能力。不断繁荣民营经济。大力开展“民营企业服务年”活动，深入落实领导包保联系民营企业制度，扩大直购电市场交易范围，增加助保金池风险补偿铺底资金，减免小微企业税负1300万元，全力推进大众创业、万众创新。

　　（六）利民惠民增福祉。坚持“把人民对美好生活的向往作为奋斗目标”，全力办好民生实事，繁荣发展社会事业，不断增强人民群众的获得感、幸福感。千方百计扩大就业。新增城镇就业4.5万人、再就业1.4万人。创建省级农民工返乡创业基地，培训农村劳动力9500人次，转移农村劳动力52万人次。发放创业担保贷款6000万元。织密扎牢社会保障救助网。新增城镇职工基本养老保险参保人员5000人、城乡居民养老保险参保人员1万人。实现城乡居民基本医疗保险制度“六统一”和吉林油田公司职工医保属地化管理。继续提高城乡低保标准。推动政府购买居家养老服务深入开展。实施“残疾人精准康复服务行动”计划。推进市社会福利院一期、市防灾减灾指挥中心和市救灾物资储备库建成使用。发放社会医疗救助和临时救助资金1050万元。稳步改善群众居住条件。深入开展“一拆三改”行动，完成国有土地上房屋征收4.2万平方米，改造棚户区房屋3000套，综合整治老旧小区20万平方米。完成80万平方米无籍房产权登记颁证。启动城东热源厂搬迁工程。改造39幢老旧楼地沟管网。铺设天然气管网5公里。优先发展教育事业。推动宁江区九年一贯制学校、回族小学教学楼和建设小学科技楼投入使用。启动供应小学教学楼新建项目。新建1所公办幼儿园。完成市特殊教育学校校园建设征地拆迁。积极支持松原职业技术学院加快发展。大力推动科技创新。落实企业研发投入财政补助、科技创新券补助等政策，培育4个市级以上科技创新中心和2个科技企业孵化器。着力提高健康服务能力。推动市中心医院异地新建项目投入使用。开工建设市妇幼保健计划生育服务中心综合楼。加快推进全民健康信息平台和医疗影像云平台建设。全力构建分级诊疗体系。大力促进医养结合。扶持发展中医药事业。全力促进文体惠民。深入开展“满族新城戏振兴工程”“地域文化创优工程”，送演出下基层271场、电影下乡13500场。深入实施全民健身计划，大力发展冰雪文化和体育产业，推动市冰上运动中心投入使用，加快市全民健身中心田径场建设。牢牢把住饮食用药安全关口。完成江南新水厂、江北净水厂扩容改造和哈达山水库取水工程，启动市城区农贸市场升级改造工程，开展“放心消费在松原”活动，推动市食品药品检验中心建成使用，实施食品安全检测7560批次、农产品农残检测4000批次。

　　（七）精准施策促脱贫。突出靶向治疗、对症下药，加大资金、项目和产业扶持力度，确保45个贫困村、19332名贫困群众告别贫困。补齐短板促脱贫。持续开展农村安全饮水改造，全面完成贫困村屯道路改造任务。争取河湖连通、节水灌溉等重点工程建设优先向贫困人口集中地区倾斜。发展产业促脱贫。扶持吉松岭、德百兴等优质合作社加快发展，带动困难群众共同致富奔小康。突出抓好贫困村与华西希望、宏远中药材种植等企业的合作，带动贫困户持续增收。积极推动上海蓝新、北京世景等光伏项目落地，依托光伏+种植、光伏+养殖，实现企业发展与农户脱贫双赢。统筹事业促脱贫。推动建档立卡贫困群众纳入低保范围。免除普通高中贫困学生学杂费用。提高贫困户新农合报销比例。发放困难家庭房屋租赁补贴1650万元。加大贫困群众就业培训力度。

　　（八）合力维稳保和谐。致力于推进治理体系和治理能力现代化，着力提高社会治理社会化、法治化、智能化、专业化水平，确保社会和谐稳定。加强社会综合治理。全面推进法治松原建设，深入开展“法律六进”等活动。抓好综合性专业人民调解中心建设。有效化解各类信访案件。依法加强宗教管理。坚决维护民族团结。突出抓好安全生产。全面落实安全生产监管责任和企业主体责任，督导企业做好安全教育培训。完善安全生产诚信体系建设，对安全生产失信行为进行联合惩戒。扎实开展油气管线等方面风险隐患排查整治，坚决遏制较大以上事故发生。有效维护公共安全。深化治安防控体系建设，深入推进“三打击一整治”专项行动和反恐怖专项斗争。全面加强流动人口和特殊人群管理。加强地震监测，扎实推进国家地震烈度速报和预警工程建设。狠抓应急管理和预案演练，有效预防和处置各类突发事件。

　　（九）改进作风提效能。“勇于自我革命”，久久为功纠正“四风”问题，加快建设人民满意的服务型政府。加大“放”的力度。落实好国家和省下放的事权，动态调整市级行政审批事项目录，严控新增目录事项。全面对标浙江“最多跑一次”改革，实行审批流程再造，精简审批程序和申报要件，压缩审批时限，提高审批效率。进一步深化商事制度改革，推动“多证合一、一照一码”广泛应用。清理和规范中介服务，破除各种服务垄断。实施减证便民专项行动，坚决砍掉各类无谓证明和繁琐手续。做精“管”的文章。严格依照权力清单、责任清单、财政专项资金清单和负面清单履职用权，坚决做到法定职责必须为。严格执行“双随机一公开”监管制度。实行联合监管和综合执法，杜绝不必要的检查抽查。启动建设松原信用网站和信息数据平台，强化联合惩戒信用监管机制。提升“服”的质量。建好政务服务“一张网”，努力构筑“互联网+政务”服务体系。加快建设网上行政审批平台，建立实体大厅、网上大厅有机结合的“线上线下”审批新机制。坚持“实”的作风。突出问题导向、目标导向和效果导向，加强督查问效，强化考核问责，做到脚踏实地、真抓实干，以发展实绩取信于民、造福于民。强化“严”的约束。严守政治纪律和政治规矩，坚决贯彻落实省委省政府和市委的决策部署。深入推进“两学一做”学习教育常态化制度化，扎实开展“不忘初心、牢记使命”主题教育。深入推进依法行政，自觉接受市人大、市政协及社会各界监督。强化审计监督，加强软环境建设，规范行政执法监管，坚决整治形式主义、官僚主义和乱作为、不作为等问题。加强政府系统党风廉政建设，从严落实“一岗双责”，扎实做好省委巡视反馈意见整改落实，持续开展扶贫领域腐败和作风问题专项治理，切实做到干部清正、政府清廉、政治清明。

　　各位代表！“使命呼唤担当，使命引领未来。”步入新时代，生态振兴、转型崛起的目标激励着我们，滚石上山、爬坡过坎的挑战考验着我们！让我们在省委省政府和市委的坚强领导下，团结协作、克难奋进，开拓创新、真抓实干，为创造松原更加美好的明天而努力奋斗！
